# Genome-Wide Identification and Characterization of *FBA* Gene Family in Polyploid Crop *Brassica napus*

**DOI:** 10.3390/ijms20225749

**Published:** 2019-11-15

**Authors:** Wei Zhao, Hongfang Liu, Liang Zhang, Zhiyong Hu, Jun Liu, Wei Hua, Shouming Xu, Jing Liu

**Affiliations:** 1 Oil Crops Research Institute of the Chinese Academy of Agricultural Sciences, Key Laboratory of Biology and Genetic Improvement of Oil Crops, Ministry of Agriculture and Rural Affairs, Wuhan 430062, China; zhaowei@caas.cn (W.Z.); wanghw@webmail.hzau.edu.cn (H.L.); zhangliang01@caas.cn (L.Z.); huzhiyong@oilcrops.cn (Z.H.); liujunocr@caas.cn (J.L.); huawei@oilcrops.cn (W.H.); 2 Henan key laboratory of Plant Stress Biology, School of Life Sciences, Henan University, Kaifeng 475004, China

**Keywords:** *Brassica napus*, aldolase, Calvin cycle, phytohormones, environmental stresses

## Abstract

Fructose-1,6-bisphosphate aldolase (FBA) is a versatile metabolic enzyme involved in multiple important processes of glycolysis, gluconeogenesis, and Calvin cycle. Despite its significance in plant biology, the identity of this gene family in oil crops is lacking. Here, we performed genome-wide identification and characterization of *FBAs* in an allotetraploid species, oilseed rape *Brassica napus*. Twenty-two *BnaFBA* genes were identified and divided into two groups based on integrative analyses of functional domains, phylogenetic relationships, and gene structures. Twelve and ten *B. napus* FBAs (BnaFBAs) were predicted to be localized in the chloroplast and cytoplasm, respectively. Notably, synteny analysis revealed that *Brassica*-specific triplication contributed to the expansion of the *BnaFBA* gene family during the evolution of *B. napus*. Various *cis*-acting regulatory elements pertinent to abiotic and biotic stresses, as well as phytohormone responses, were detected. Intriguingly, each of the *BnaFBA* genes exhibited distinct sequence polymorphisms. Among them, six contained signatures of selection, likely having experienced breeding selection during adaptation and domestication. Importantly, *BnaFBAs* showed diverse expression patterns at different developmental stages and were preferentially highly expressed in photosynthetic tissues. Our data thus provided the foundation for further elucidating the functional roles of individual *BnaFBA* and also potential targets for engineering to improve photosynthetic productivity in *B. napus*.

## 1. Introduction

Fructose-1,6-bisphosphate aldolase (FBA, EC4.1.2.13 or aldolase) is an essential metabolism enzyme in the glycolytic pathway [1]. FBA catalyzes the reversible aldol cleavage of fructose-1,6-bisphosphate (FBP) into dihydroxyacetone phosphate (DHAP) and glyceraldehyde-3-phosphate (G3P), two important intermediates for oil biosynthesis [2]. DHAP could be further converted to diacylglycerol (DAG) by multiple enzymatic reactions, and DAG is a key substrate of diacylglycerol acyltransferase (DGAT) for the synthesis of triacylglycerols (TAGs). Meanwhile, G3P could be converted to malonyl CoA (coenzyme A) that is then used to produce fatty acids. Thus, FBA is not only one of the key regulatory enzymes in the glycolysis pathway but also may control the flux of carbohydrates and, therefore, play an important role in the oil yield of oilseeds [3,4].

FBAs could be broadly classified into two classes, namely class-I and class-II, based on their catalytic mechanisms and prevalence among species in evolution [5,6]. Class-I FBAs are usually tetrameric enzymes, forming a Schiff base with the substrate as intermediate, and utilize a lysine residue to generate a nucleophilic enamine from DHAP and are not inhibited by EDTA or affected by potassium ions. Class-II FBAs are found as homodimers and require divalent cations as cofactors to stabilize the DHAP enolate intermediate involved in the aldol condensation reaction and are inhibited by EDTA [7]. FBAs of class-I are found in some bacteria, animals, and plants, while class-II FBAs occur in most bacteria, yeast, and fungi [8,9,10]. However, FBAs of class-II are also found in wheat and green algae, such as *Euglena gracilis*, *Chlamydomonas mundane*, and *Chlamydomonas rheinhardii* [11,12,13].

In higher plants, one set of FBA isoenzymes is localized in the cytosolic (cFBA), and another one in the chloroplast/plastid (cpFBA) [14,15]. Both cFBA and cpFBA are encoded by separate nuclear genes that probably evolved from duplication of a common ancestral gene [16]. To date, different members of the *FBA* family genes have been reported in a variety of monocots and eudicots species. For example, in *Arabidopsis thaliana* (*A. thaliana*), eight *FBA* family genes (*AtFBA1–8*) were identified, including three chloroplast/plastid members (*AtFBA1–3*) and five cytosolic members (*AtFBA4–8*) [17]. In tomato, eight *FBA* genes, including five cFBAs and three cpFBAs, were characterized based on the phylogenetic tree, gene structures, and conserved motifs [18,19]. In addition, two homologous genes of cpFBAs were isolated from green leaves of *Nicotiana paniculata* [20]. One *SpFBA* gene was cloned from *Sesuvium portulacastrum* roots [21]. In wheat, 21 genes encoding *Thermus aquaticus* FBA (TaFBA) isoenzymes were identified and categorized into three subgroups of class-I *cpFBAs*, class-I *cFBAs*, and class-II *cpFBAs* [11]. In rice, two *OsFBAs* were reported to localize in the chloroplasts, while the other five *OsFBAs* in the cytoplasm [22]. One chloroplastic *FBA* and one cytosolic *FBA* were detected in the leaves of maize seedlings [23]. Four developmentally up-regulated *FBAs* (*CoFBA1–4*) genes were identified in tea oil tree (*Camellia oleifera*) seeds [24]. However, there is no report on the identification of *FBA* family genes so far in oilseed rape *Brassica napus*.

Recent studies suggest that the *FBA* genes are playing important roles in diverse significant physiological and biochemical processes in plants. The cpFBA is an essential enzyme in the Calvin cycle, in which its activity generates metabolites for starch biosynthesis [25]. For instance, the loss of the *AtFBA6* function resulted in a lower germination rate after abscisic acid (ABA) treatment in *A. thaliana* [17]. The expression levels of *CoFBA1* and *CoFBA3* genes were highly correlated with the amount of tea oil in the seeds of tea oil tree [24]. Reduction of plastid FBA activity inhibits photosynthesis and alters carbon partitioning in potato, whereas increased FBA activity in plastids could promote CO_2_ fixation and enhance the growth and photosynthesis in tobacco [26,27]. Inhibition of chloroplastic FBA affects the development of fruit size in tomato [28]. Gibberellin (GA) treatment could increase the levels of cytoplasmic *FBA* in all regions of the roots, resulting in the stimulation of the root growth mediating the energy production in rice [29]. Thus, *FBA* family genes appear to be also associated with the response to phytohormones. Besides, *FBAs* also participate in response to various environmental stimuli in plants, such as salinity, drought, anoxygenic stress, abnormal temperature, light acclimation, and *Rhizoctonia solani Kuhn* infection [21,30,31,32].

*Brassica napus* L. (*B. napus*), a relatively recent allotetraploid formed from hybridization between *Brassica rapa* (*B. rapa*) and *Brassica oleracea* (*B. oleracea*), is the second-largest source of vegetable oil crop and cultivated around the world [33,34]. *B. napus* has adapted to diverse climate zones and latitudes by forming three main ecotype groups, namely winter, semi-winter, and spring types [35,36]. Although *FBA* family genes have been studied in several plant species, little is known regarding this gene family in oil crops. Here, we systematically identified *FBA* family genes in *B. napus* and profiled their gene structures, chromosome locations, conserved motifs, *cis*-acting elements in the promoter regions, phylogenetic classifications, and sequence polymorphisms. We further analyzed their expression patterns in different developmental tissues and in response to stresses. Our results provided the foundation for further elucidating the functional roles of *FBA* family genes and potential targets for engineering to improve photosynthetic capacity in *B. napus*.

## 2. Results

### 2.1. Identification, Properties, and Genomic Distribution of BnaFBA Genes

To identify each member of the *FBA* gene family in *B. napus*, the glycolytic domain (PF00274) and fructose-bisphosphate aldolase class-II domain (PF01116) from the Pfam database (http://pfam.xfam.org/) were employed as queries to search against *B. napus* var. Darmor-*bzh* protein dataset. The peptides of putative *B. napus* FBAs (BnaFBAs) with the best hit of *A. thaliana* FBAs (AtFBAs) and TaFBAs were further used to predict functional domains by using the Pfam and SMART databases to confirm the presence of the fructose-bisphosphate aldolase domains. As indicated in Table 1, we identified 22 *BnaFBA* genes in *B. napus*. Meanwhile, we found 14 *BraFBAs* and fourteen *BolFBAs* in *B. rapa* var. Z1 and *B. oleracea* var. HDEM, respectively (Table S1). 

The transcript length of *BnaFBA* genes varied from 951 bp to 4149 bp. All identified *BnaFBA* genes encoded proteins ranging from 317 (*BnaFBA2e*) to 1382 (*BnaFBA9b*) amino acids (aa), molecular weight (MW) from 34.18 (*BnaFBA2e*) to 148 kDa (*BnaFBA9b*), isoelectric point (pI) from 5.7 (*BnaFBA2a*) to 8.47 (*BnaFBA3a*), and grand average of hydropathy (GRAVY) from −0.263 (*BnaFBA3a*) to 0.076 (*BnaFBA9b*) (Table 1). Twelve BnaFBAs (BnaFBA1a/b/c, BnaFBA2a/b/c/d/e/f, BnaFBA3a/b, and BnaFBA9a) were predicted to be localized in chloroplast, with the other 10 BnaFBAs (BnaFBA5a/b, BnaFBA6, BnaFBA8a/b/c/d/e/f, and BnaFBA9a) being in the cytoplasm (Table 1). Similarly, in *B. rapa* and *B. oleracea*, seven *B. Oleracea* FBAs (BolFBAs) and seven *B. rapa* FBAs (BraFBAs) were predicted to be chloroplast-localized proteins, six BolFBAs and six BraFBAs were cytoplasm-localized ones, whereas BolFBA9 and BraFBA6a were predicted to be localized in plasma membrane and nucleus, respectively (Table S1).

Based on the functional domains contained, the 22 *BnaFBAs* could be divided into two classes—class-I and class-II. All the BnaFBAs of class-I harbored one glycolytic domain (PF00274) across the proteins, and BnaFBA9a and BnaFBA9b of class-II had one fructose-bisphosphate aldolase class-II domain (PF01116). Except for the glycolytic domain (PF00274), some members of class-I FBA, such as BnaFBA5b, as the longest FBA of class-I with 870 aa, also harbored PRK (phosphoribulokinase kinase) (PF00485) and UPRTase (uracil phosphoribosyltransferase) (PF14681) domains in the N terminal. Likewise, in *B. oleracea*, BolFBA5 contained three functional domains of PF00274, PF00485, and PF14681, and BolFBA6b had two tandem copies of glycolytic domains (PF00274). Interestingly, BraFBA6a contained both glycolytic domain (PF00274) and RNA polymerase II-binding domain (PF04818), suggesting that it might be involved in RNA processing in the nucleus (Table S1).

**Figure 1:**
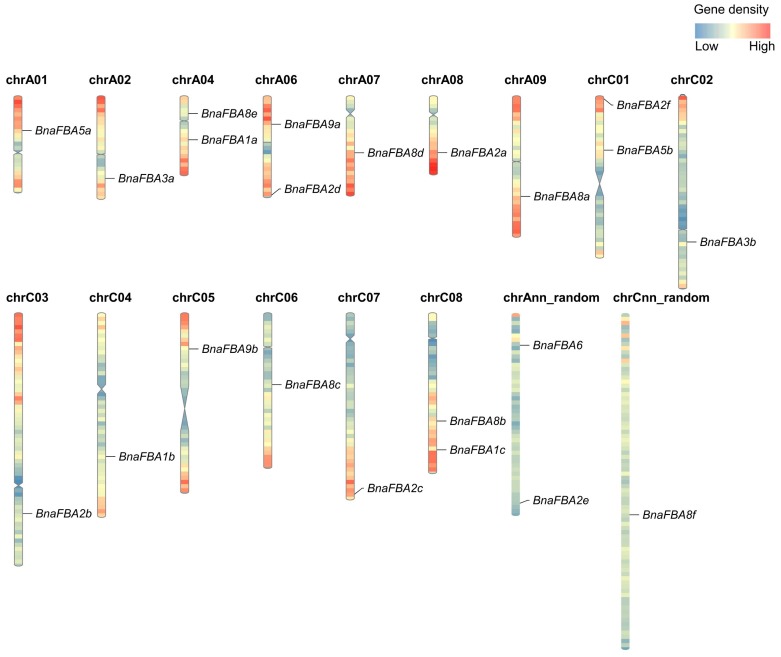
Genomic distributions of *BnaFBA* genes on *B. napus* chromosomes. The *BnaFBAs* were plotted based on the location of genes, length of chromosomes, and positions of centromeres. Heatmap of each chromosome indicated the gene density by the frequency per 1 Mb.

To attain a general view of the distribution of *BnaFBA* genes on the genome of *B. napus*, the 22 *BnaFBAs* were mapped on the corresponding chromosomes according to their physical positions. Of the 22 *BnaFBA* genes, 19 were evenly distributed on the 15 *B. napus* chromosomes, while the three other *BnaFBAs* were assigned to the random chromosomes (two on the An chromosomes and one on the Cn chromosome). The number of *BnaFBAs* per chromosome ranged from one to two. Among them, eleven *BnaFBA* genes were distributed over the seven A subgenome chromosomes of *B. napus*, including A01, A02, A04, A06, A07, A08, and A09, as well as Ann_random. Equally, the other 11 *BnaFBAs* were distributed over eight C subgenome chromosomes of *B. napus*, including C01, C02, C03, C04, C05, C06, C07, and C08, as well as Cnn_random. Most of the *BnaFBAs* were not localized in the terminal regions of the chromosomes, where the gene density was relatively high in *B. napus* (Figure 1).

### 2.2. Phylogenetic and Structure Analysis of BnaFBAs

**Figure 2:**
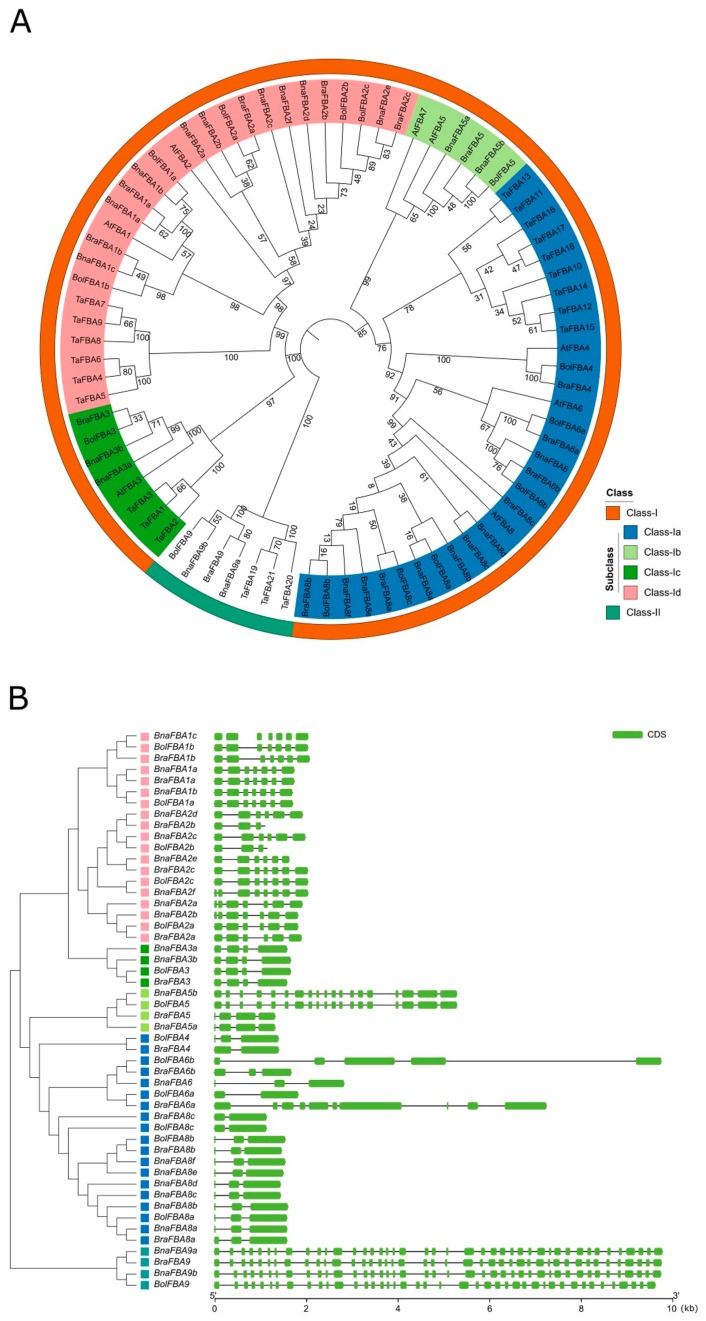
Phylogenetic relationships and gene structure of *BnaFBA* genes. (**A**) Phylogenetic relationships of *BnaFBAs*, *BraFBAs*, *BolFBAs*, *AtFBAs,* and *TaFBAs*. The unrooted tree was generated using MEGA7.1 software by the neighbor-joining method. The numbers next to the branch show the 1000 bootstrap replicates expressed in percentage. The phylogenetic classes of *FBA* genes were marked by corresponding colors that are shown in the color legend at the bottom right. (**B**) The schematic diagrams of the exon-intron organization of *FBA* genes in *B. napus*, *B. rapa,* and *B. oleracea*. The phylogenetic tree of the *FBA* genes is placed at the left, and the color squares represent phylogenetic classes. The green boxes and lines indicate CDS (coding sequence) and introns, respectively. The length of the scale is at the bottom.

To explore the molecular evolution of the *FBA* gene family in *B. napus*, a total of 79 *FBA* genes from *B. napus*, *B. rapa*, *B. oleracea*, *A. thaliana,* and wheat, were used to construct an unrooted phylogenetic tree. According to the phylogenetic relationships of these *FBA* genes, they could be divided into two independent classes, consistent with the classification by functional domains they contained (Figure 2A). Furthermore, the *FBA* genes of class-I could be further classified into four subclasses, namely class-Ia, class-Ib, class-Ic, and class-Id. Therefore, in *B. napus*, there were seven *BnaFBA* genes in the class-Ia group, two in class-Ib, nine in class-Ic, two in class-Id, and two in class-II. Consistent with its polyploid origin, except for the FBA4 gene, the genome of *B. napus* maintained homologs of each *FBA* gene derived from the diploid parents, *B. rapa* and *B. oleracea* (Figure 2A).

Based on the gene information of the genome available in the GENOSCOPE database, we performed gene structure analysis by comparing the coding sequence (CDS) of *BnaFBAs, BraFBAs,* and *BolFBAs*. In *B. napus*, A subgenome homologs and C subgenome homologs of *FBA* genes that were in the adjacent branches of the phylogenetic tree exhibited the same gene structure (Figure 2B). Within each class of *FBA* genes, the features of exons, such as order, length, and number, were largely conserved except for *FBA6* genes (Figure 2B). Besides, the organization of the introns of *BnaFBA* genes was highly variable. The length of introns varied extensively in different members of the *BnaFBA* gene family, ranging from 30 to 4152 bp, with the number ranging from 2 to 41. Compared to the class-I *BnaFBA* genes, the class-II genes were much longer and had much more exons and introns (Figure 2B).

**Figure 3:**
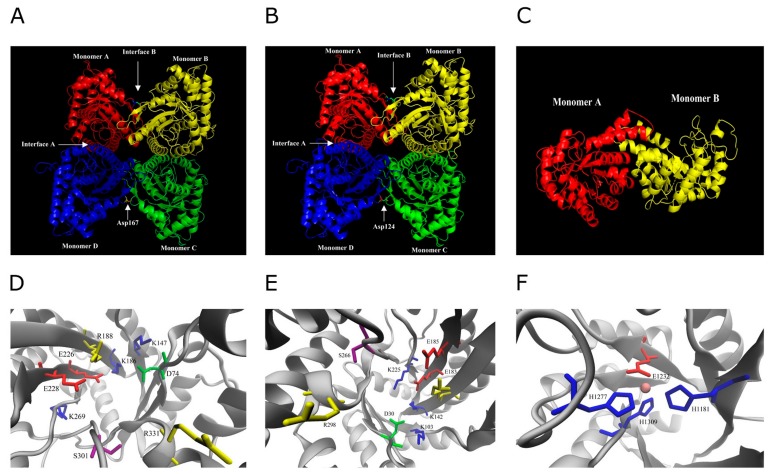
Predicted three-dimensional model of representative *Brassica napus* Fructose-1,6-bisphosphate aldolase (BnaFBA) proteins. (**A**) Tetrameric BnaFBA1a. (**B**) Tetrameric BnaFBA8a. (**C**) Dimeric BnaFBA9a. (**D**) Active site residues of BnaFBA1a. (**E**) Active site residues of BnaFBA8a. (**F**) Active site residues of BnaFBA9a. The sites of the Asp167/124 substitutions are indicated on interface B, and the filled circle represents the divalent metal cation of BnaFBA9a.

To further explore the higher-order structure of the BnaFBA proteins, the three-dimensional (3D) structural models for BnaFBA1a, BnaFBA8a, and BnaFBA9a were generated using SWISS-MODEL. Based on the experimental structure of class-I rabbit muscle aldolase, the SWISS-MODEL analysis results revealed that BnaFBA1a and BnaFBA8a of the class-I group could form tetramers structures, and interfaces A and B were observed in the BnaFBA1a and BnaFBA8a tetramers. Different from BnaFBA1a and BnaFBA8a, BnaFBA9a belonged to class-II group and form dimers based on its relatively high similarity to class-II aldolases of *Thermus aquaticus* (Figure 3A–C). Furthermore, the catalytic residues of D74-K147-K186-R188-E226-E228-K269-S301-R331 and D30-K103-K142-R144-E183-E185-K225-S266-R298 were observed in BnaFBA1a and BnaFBA8a, respectively. Additionally, similar to the class-II FBAs of *Thermus aquaticus*, BnaFBA9a contained active sites of H1181-E1232-H1277-H1309 that also serve as the divalent metal cation binding sites (Figure 3E,F). Multiple sequence alignment results indicated that class-I BnaFBAs, BraFBAs, BolFBAs, and AtFBAs had high conserved catalytic residues (Figure S1). The active sites among class-II BnaFBAs, BraFBAs, and BolFBAs were the same as FBA isozymes in the *Thermus aquaticus* (Figure S2).

### 2.3. Synteny and Gene Duplication of BnaFBA Genes

**Figure 4:**
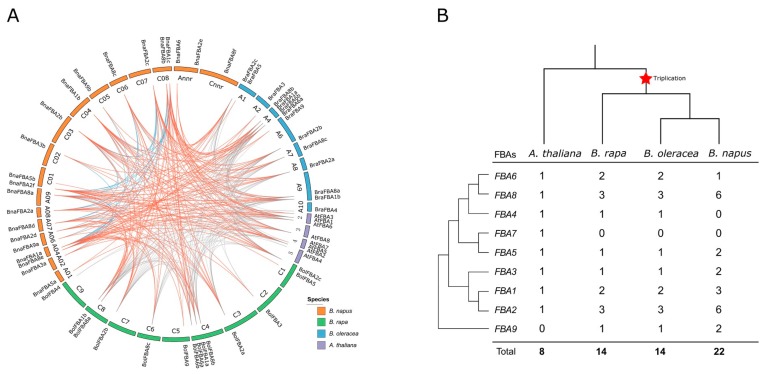
Collinear correlations and copy number variation of the *FBA* family genes in *B. napus*, *B. rapa*, *B. oleracea,* and *A. thaliana*. (**A**) Collinear correlations of FBA genes in the *B. napus*, *B. rapa*, *B. oleracea,* and *A. thaliana* genomes. The *B. napus*, *B. rapa*, *B. oleracea,* and *A. thaliana* chromosomes were colored by corresponding colors that are shown in the color legend at the bottom right. The blue lines represent the collinear correlations of FBA genes within *B. napus,* and the orange lines are for the collinear correlations of *FBA* genes between *B. napus* and the other species, with the grey lines representing the collinear correlations of *FBA* genes among *B. rapa*, *B. oleracea,* and *A. thaliana*. The figure was created using CIRCOS software. (**B**) Copy number variation of the *FBA* family genes in *B. napus*, *B. rapa*, *B. oleracea,* and *A. thaliana*. The phylogenetic tree of *FBA* genes is shown on the left, with the species tree shown at the top. The *Brassica*-specific triplication was indicated on the branches of the trees according to the Plant Genome Duplication Database. The numbers are the copy numbers of each FBA gene in *A. thaliana*, *B. napus*, *B. rapa,* and *B. oleracea*.

*A. thaliana* is the most prominent model system for plant molecular biology and genetics research, whose structural genes have been identified and functionally characterized. Thus, we traced the orthologous gene pairs between *A. thaliana* and *Brassica* species to investigate the evolutionary history by syntenic gene analysis. A total of 29 orthologous gene pairs were identified between *A. thaliana* and *B. napus*, 20 between *A. thaliana* and *B. rapa*, and 19 between *A. thaliana* and *B. oleracea*. In addition, we also obtained 29, 15, and 15 paralogous gene pairs within *B. napus*, *B. rapa,* and *B. oleracea*, respectively (Figure 4A). The previous study revealed that crucifer (*Brassicaceae*) lineage experienced two whole-genome duplications (named α and β) and one triplication event (γ) shared by most dicots [37]. Moreover, *Brassica* species experienced an extra whole-genome triplication (WGT) event compared with *A. thaliana* [38]. As WGT of the *Brassica* ancestor, *FBA* genes in the *A. thaliana* genome might have triplicated orthologous copies in *B. rapa* and *B. oleracea*. Consequently, some *FBA* genes (i.e., *FBA2* and *FBA8*) existed triple the number of those in *A. thaliana*, while the other genes (i.e., *FBA1*, *FBA3*, *FBA5,* and *FBA6*) had double or equal the number (Figure 4B). The *FBA* genes of *B. napus* were inherited from its diploid ancestors; thus, most of the *BnaFBA* genes were double the number of those in *B. rapa* and *B. oleracea* (i.e., *FBA2*, *FBA3*, *FBA5*, *FBA8,* and *FBA9*). However, both *FBA1* and *FBA6* genes lost one copy in the C subgenome of *B. napus*, while FBA4 lost all the copies in *B. napus* compared to its two ancestors (Figure 4B). Gene duplication analysis with syntenic and phylogenomic approaches using tool DupGen_finder in *B. napus* showed that all *BnaFBA* genes had corresponding duplicate genes. In *B. napus*, a total of 42 gene pairs were derived from whole-genome duplication (WGD), with one gene pair of *BnaFBA2e*-*BnaFBA2f* being derived from transposed duplication (Table S2).

### 2.4. Cis-Acting Elements in the Putative Promoter Regions of BnaFBA Genes

As important molecular switches, *cis*-acting elements in the promoter region could provide useful information to understand the function and regulation of the genes during plant development and responses to various stresses. The 1.5 kb genomic DNA sequences identified from upstream of the *BnaFBA* genes were extracted and deployed in *cis*-acting regulatory elements analysis with PlantCARE. Various *cis*-acting regulatory elements existed within the promoter regions of *BnaFBA* genes (Table 2 and Table S3). For example, *BnaFBA* genes contained multiple phytohormone responsive elements, such as ABRE (abscisic acid-responsive element), AuxRE (auxin-responsive element), ERE (ethylene-responsive element), GARE (gibberellin-responsive element), MeJARE (MeJA-responsive element), and SARE (salicylic acid-responsive element). This suggested that the expression of *BnaFBAs* might be induced by different phytohormones. Besides, the *cis*-acting regulatory elements involved in stress-responsive elements, such as ARE (anoxic-responsive element), DRE (damage-responsive element), DIRE (drought-responsive element), DSRE (drought- and stress-responsive element), HSRE (heat stress-responsive element), LTRE (low-temperature-responsive element), and WRE (wound-responsive element), were also found within the promoters of *BnaFBA* genes, suggesting that expression levels of *BnaFBAs* might be also regulated by various environmental factors like drought, heat, and low-temperature. Globally, three phytohormone-related elements (i.e., ABRE, ERE, and MeJARE) and two stress-responsive elements (ARE and HSRE) were detected with high frequency in the promoter regions of *BnaFBA* genes. Notably, each *BnaFBA* had multi-copy LREs (light-responsive elements) ranging from 8 to 26, implying that *BnaFBAs* might play roles in light responses.

### 2.5. Natural Variations of BnaFBA Family Genes in B. Napus

**Figure 5:**
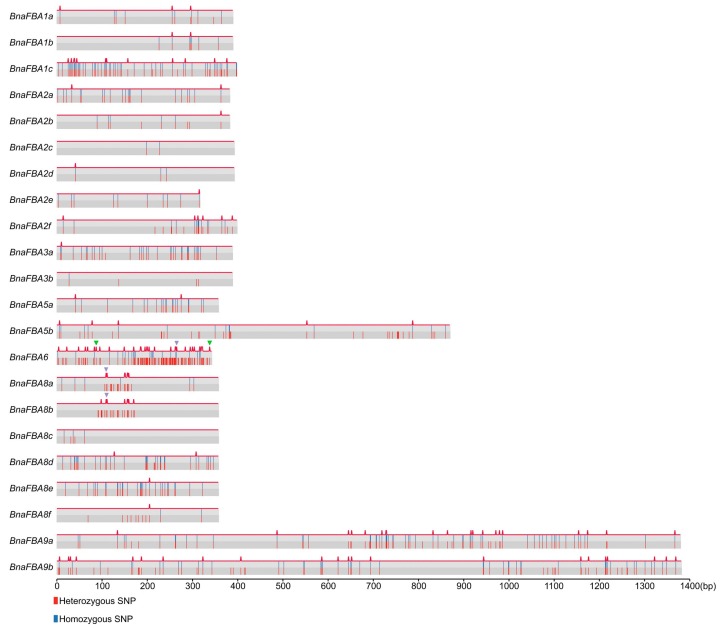
The variation distribution of *BnaFBA* genes. The heterozygous SNPs and homozygous SNPs were marked as red and blue lines, respectively. The red peak on the top of each *FBA* gene represents nonsynonymous variation. The green and purple triangles signify frameshift deletion/insertion and stop-gain variations, respectively. The length of the scale is placed at the bottom.

Critical sequence polymorphism across the gene and its flanking regions may reflect the evolutionary process of species adapting to different environments. The public resequencing datasets of 991 *B. napus* germplasm accessions covering three main ecotype groups, namely winter, semi-winter, and spring types, were collected for variation analysis of the *BnaFBA* family genes [39]. The polymorphism sites of CDS in *BnaFBA* family genes ranged from two (*BnaFBA2c*) to 130 (*BnaFBA6*) (Table 3). The π and θ_w_ of nucleotide diversity parameters extended from 0.00057 (*BnaFBA3b*) to 0.04158 (*BnaFBA6*) and from 0.00054 (*BnaFBA3b*) to 0.02255 (*BnaFBA6*), respectively. Some members of *BnaFBA* gene family were conserved, such as *BnaFBA3b* (θ_w_ = 0.00054), *BnaFBA2c* (θ_w_ = 0.00092), and *BnaFBA2d* (θ_w_ = 0.00098), while others had high polymorphism, such as *BnaFBA9b* (θ_w_ = 0.00511), *BnaFBA5a* (θ_w_ = 0.00535), *BnaFBA8b* (θ_w_ = 0.0056), *BnaFBA8e* (θ_w_ = 0.00598), *BnaFBA3a* (θw = 0.00665), *BnaFBA8a* (θ_w_ = 0.00706), *BnaFBA1c* (θ_w_ = 0.00762), *BnaFBA8d* (θ_w_ = 0.00807), and *BnaFBA6* (θ_w_ = 0.0225). Besides, the *BnaFBA6* variation ratio reached a peak of 12.60 among the 22 *BnaFBA* genes, whereas *BnaFBA2c* had the lowest variation ratio of 0.17, with only two polymorphic sites (Table 3). Generally, due to the longer length of genes, *BnaFBA9a* and *BnaFBA9b* of class-II had much more variations than *FBA* genes of class-I; however, the variation ratio and nucleotide diversity of the coding regions of *BnaFBA9a* and *BnaFBA9b* showed no difference with the *BnaFBA* genes of class-I except for *BnaFBA6* (Table 3). In addition to CDS regions, a total of 1029 and 814 variations were also identified in the upstream/downstream 1.5 kb regions and intronic regions of *BnaFBA* genes, respectively (Table S4). Notably, *BnaFBA8a*, *BnaFBA8b,* and *BnaFBA6* each harbored one stop-gain mutation that led to premature stop codons, which indicated that these genes exhibited loss-of-function (Figure 5, Table S5). Only a few of the Indels had been detected in *BnaFBAs*. For example, *BnaFBA1c* had three non-frameshift Indels, and *BnaFBA3b* contained only one non-frameshift Indel, with *BnaFBA6* harboring two frameshift deletion/insertion variations (Figure 5). To study the population selection pressure, we conducted neutral testing using Tajima’s D. Tajima’s D values of all the *BnaFBAs* were positive, with significant Tajima’s D values (*p* < 0.01) being observed in *BnaFBA1a*, *BnaFBA1e*, *BnaFBA2d,* and *BnaFBA2e* (Table 3). Particularly, the Tajima’s D values of *BnaFBA1b* and *BnaFBA8a* reached extremely significant levels (*p* < 0.001 and *p* < 0.0001). Based on the variation of *BnaFBA* genes, we performed principal component analysis (PCA), in which no significant difference in the polymorphism of *BnaFBA* genes was seen between different ecotype groups of *B. napus* (Figure S3).

### 2.6. Expression Patterns of BnaFBA Genes

The members of the *FBA* gene family are playing important roles in diverse significant physiological and biochemical processes in plants [21,25]. Besides, FBAs also participate in response to various environmental stimuli [30,31,32]. To identify the function of *BnaFBA* genes under various conditions, the expression levels of *BnaFBAs* were evaluated during growth and development, as well as in response to biotic stresses and phytohormones in *B. napus*.

**Figure 6:**
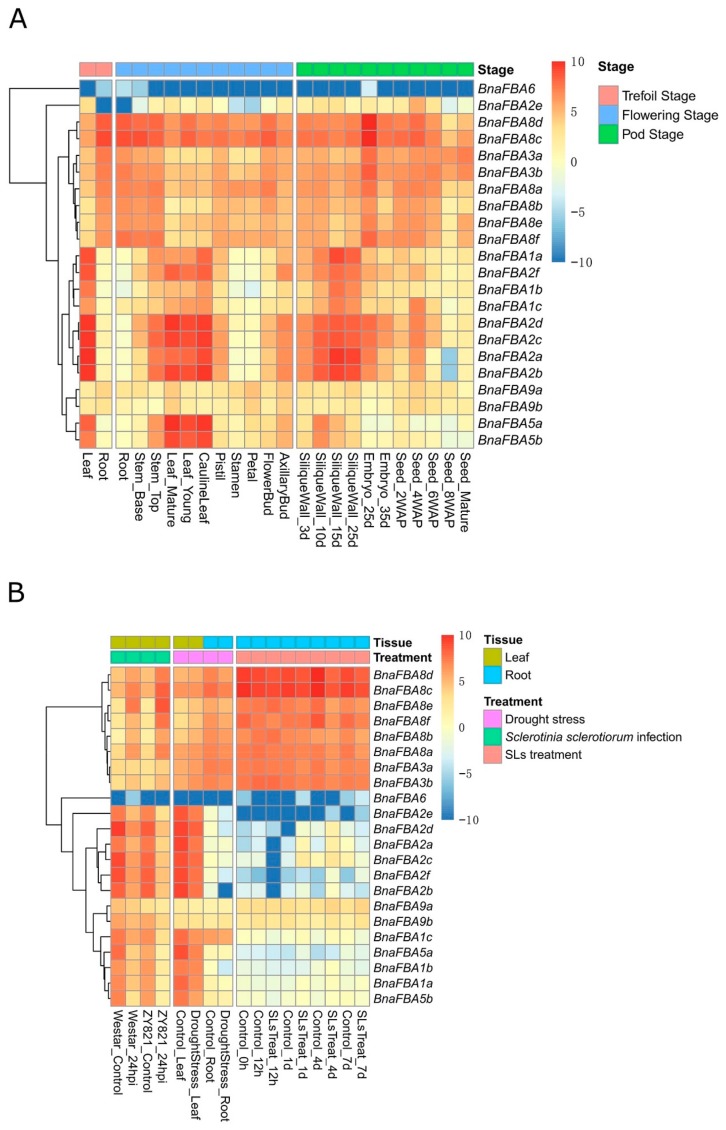
Expression analysis of the 22 *BnaFBA* genes in *B. napus*. (**A**) Expression patterns of the *BnaFBA* genes in various tissues at different developmental stages. The color bar represents log2 expression levels (FPKM, fragments per kilobase of exon per million fragments mapped) of each gene. (**B**) Expression patterns of the *BnaFBA* genes under drought stress, *Sclerotinia sclerotiorum* infection, and strigolactones treatments in *B. napus*. The color scale of heatmap indicates expression values, with blue-white and red representing low and high levels of transcript abundance, respectively. The cluster tree of *BnaFBA* genes, based on the expression level, is shown on the left.

To explore the expression patterns of the *BnaFBAs* during growth in *B. napus*, we analyzed their expression levels in twelve various tissues (leaf, root, stem, cauline leaf, pistil, stamen, petal, flower bud, axillary bud, silique wall, embryo, and seed) at different developmental stages. As a result, at the trefoil stage and the flowering stage, *BnaFBA* genes showed opposite expression patterns between leaves and roots. For example, *BnaFBA1a/b/c*, *BnaFBA2a/b/c/d/f,* and *BnaFBA5a/b* displayed relatively high expression level in leaves than that in roots. On the contrary, *BnaFBA3a/b* and *BnaFBA8a/b/c/d/e/f* exhibited relatively high expression levels in roots than that in leaves. At the flowering stage, the expression levels of *BnaFBA1a/b/c* and *BnaFBA2a/b/c/d/f* were higher in pistil than that in stamen and petal tissues. At the pod stage, *BnaFBA1a/b/c* and *BnaFBA2a/b/c/d/f* were highly expressed in the silique wall, with their expression levels being increased during the development of pod. *BnaFBA3a*, *BnaFBA3b*, *BnaFBA8c,* and *BnaFBA8d* were highly and constantly expressed in all tissues, particularly showing the highest expression levels in the embryo at 25 days after pollination (DAP). In addition, the expression of *BnaFBA2a/b/c/d/e/f* increased during the development of seeds and reached high levels at 4 weeks after pollination (WAP), then decreased in the following mature stages of seed development (Figure 6A). *BnaFBA9a* and *BnaFBA9b* of class-II showed constitutive expression at relatively low levels in all the tissues examined. *BnaFBA6* of class-I exhibited unexpressed in most tissues. Taken together, these results suggested that *BnaFBA* genes displayed diverse spatiotemporal expression patterns during the growth and development of different tissues in *B. napus*. 

To identify the physiological functions of *BnaFBAs* in response to various environmental stresses and phytohormones, as an illustration, we investigated the expression of *BnaFBA* genes in leaf and root tissues under drought stress, *Sclerotinia sclerotiorum* infection, and strigolactones (SLs) treatments. After 24 h of *Sclerotinia sclerotiorum* infection, *BnaFBA1a/b/c*, *BnaFBA2a/b/c/d/e/f*, *BnaFBA5a/b,* and *BnaFBA9b* were down-regulated, while *BnaFBA3a/b* and *BnaFBA8a/b/c/d/e/f* were up-regulated compared to the control in the leaves of the two *B. napus* cultivars—wester and ZY821 (Figure 6B). Under drought stress, the expression levels of *BnaFBA1a/c*, *BnaFBA2a/b/c/d/e/f,* and *BnaFBA5a/b* decreased in the leaves. Similarly, the expression levels of *BnaFBA1b*, *BnaFBA2b/d/e/f* also decreased in roots compared to the control (Figure 6B). We further examined the expression of *BnaFBAs* under exogenous SLs treatments in *B. napus*. Notably, the members of the *BnaFBA2* group showed diverse expression patterns after SLs treatments in *B. napus*. At 12 h after SLs treatments, the expression levels of *BnaFBA2a/b/c/f* were significantly down-regulated, while the expression of *BnaFBA2d* was slightly decreased relative to the control. At 1 day after SLs treatment, *BnaFBA2a/b/c/d* expression levels were significantly up-regulated compared to the control, whereas *BnaFBA2e/f* showed no difference. At 4 days after SLs treatment, all members of the *BnaFBA2* group exhibited up-regulated expression compared to the control. Then, at 7 days after SLs treatments, the expression levels of *BnaFBA2a/b/c/d* turned to be down-regulated, while *BnaFBA2e/f* expression levels were still up-regulated compared to the control (Figure 6B).

### 2.7. qRT-PCR Analysis of Selected BnaFBA Genes under Abiotic Stresses

**Figure 7:**
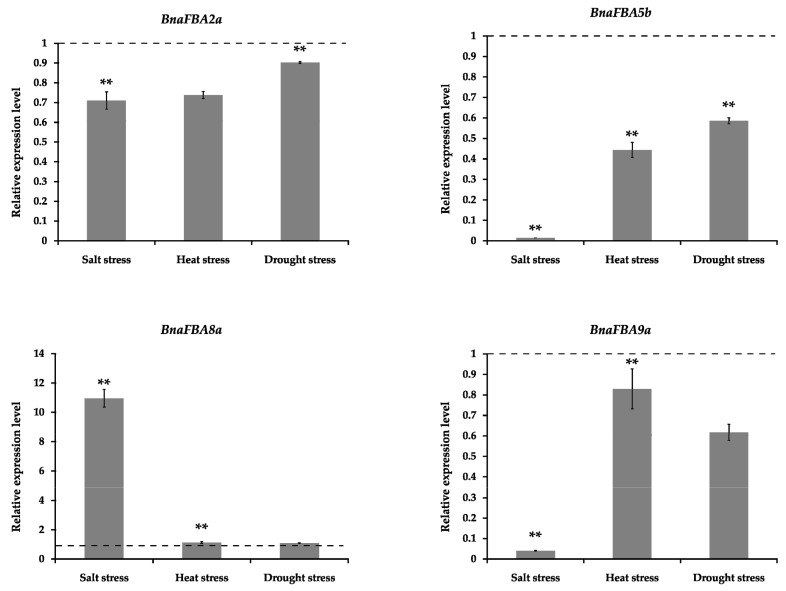
The real-time PCR analysis of the selected four representative *BnaFBA* genes responded to salt, heat, and drought stresses. The dotted lines represent the equivalent levels of expression. Statistically significant differences (Student’s *t*-test) are indicated as followed: ** *p* < 0.01.

To further validate the functional roles of *BnaFBAs* under abiotic stresses, four *BnaFBA* genes from different clusters were selected for the examination of their expression levels under three stress conditions using quantitative real-time PCR (qRT-PCR) in *B. napus*. These genes included *BnaFBA2a*, *BnaFBA5b,* and *BnaFBA8a* of class-I, and *BnaFBA9a* of class-II. The qRT-PCR analysis was carried out using rapeseed plants exposed to salt, heat, and drought stresses. At 3 days after NaCl treatment, the expression level of *BnaFBA8a* was significantly up-regulated by approximately 11-fold compared with the control, while *BnaFBA2a*, *BnaFBA5b,* and *BnaFBA9a* were significantly down-regulated, particularly, the expression levels of *BnaFBA5b* and *BnaFBA9a* were largely reduced (Figure 7). In addition, at three days after heat and drought stress treatments, the expression level of *BnaFBA5b* was significantly reduced nearly by half, whereas the expression of *BnaFBA8a* was slightly increased compared to the control (Figure 7).

### 2.8. Co-Regulatory Networks of BnaFBA Genes

**Figure 8:**
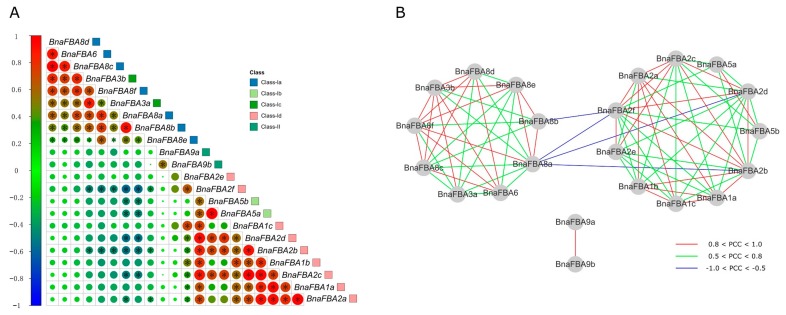
Correlations and co-regulatory networks of *BnaFBA* genes. (**A**) Correlation analysis of *BnaFBA* genes was performed based on the Pearson correlation coefficients (PCCs) of *BnaFBA* gene pairs. Correlations are indicated by the size and color of circles. The left bar represents the correlation values of PCCs. The class information for *BnaFBAs* is indicated by the squares with different colors at the right. Black star signifies the correlation with *p*-value ≤ 0.05. (**B**) The co-regulatory network of *BnaFBA* genes was generated on the basis of the significant PCCs of gene pairs (*p*-value ≤ 0.05). The distinct correlation levels of gene pairs are marked by edge lines with different colors shown at the bottom. The co-regulatory network was illustrated by Cytoscape (version 3.1, Seattle, WA, USA).

Based on the publically available RNA-seq datasets collected from different tissues, biotic and abiotic stresses, and phytohormone treatments in *B. napus*, we calculated the Pearson correlation coefficients (PCCs) of the expression levels of *BnaFBA* genes and constructed co-regulatory networks. Positive correlations were observed between members of class-Ia and class-Ib, such as *BnaFBA3a/b*, *BnaFBA8a/b/c/d/e/f,* and *BnaFBA6*. Likewise, the *BnaFBA* genes of class-Ic and class-Id also showed positive correlations between each other. Particularly, members of class-Id, including *BnaFBA1a/b/c* and *BnaFBA2a/b/c/d*, exhibited strong positive correlations. *BnaFBA2b* and *BnaFBA2f* showed significant negative correlation with *BnaFBA3a/b* and *BnaFBA8a/b/e/f*. However, they displayed a significant positive correlation with other *BnaFBA* genes of class-Ic and class-Id (Figure 8A). All the significant PCCs (*p*-value ≤ 0.05 and |PCC| > 0.5) of *BnaFBAs* were extracted and used to construct co-regulatory networks delineated by Cytoscape (version 3.1, Seattle, WA, USA) (Figure 8B). The co-regulatory networks of *BnaFBAs* were constituted with 22 nodes and 83 edges. There were four *BnaFBA* gene pairs showing negative correlations (*p*-value ≤ 0.05 and PCC < −0.5), including *BnaFBA2f* and *BnaFBA8a*, *BnaFBA2b* and *BnaFBA8a*, *BnaFBA2f* and *BnaFBA8b*, and *BnaFBA2d* and *BnaFBA8a*. Besides, 79 *BnaFBA* gene pairs showed positive correlations, of which 38 pairs exhibited strong positive correlations (*p*-value ≤ 0.05 and PCC > 0.8). Notably, three gene clusters of *BnaFBAs* were observed, including cluster 1 consisting of *BnaFBA3a/b*, *BnaFBA6,* and *BnaFBA8a/b/c/d/e/f*, cluster 2 consisting of *BnaFBA1a/b/c*, *BnaFBA2a/b/c/d/e/f,* and *BnaFBA5a/b,* and cluster 3 consisting of *BnaFBA9a/b*. Members within each cluster showed strong positive correlations between the expression levels, whereas only a few significant negative correlations existed between cluster 1 and cluster 2. Moreover, cluster 3 was independent among the three clusters (Figure 8B).

## 3. Discussion

The Calvin cycle is the initial pathway of photosynthetic carbon fixation, which plays a requisite role in the growth and development of plants [40]. Extensive efforts for seeking a breakthrough in the regulation of this cycle have been made to substantially enhance photosynthetic CO_2_ capacity and plant productivity. The carboxylation capacity of ribulose bisphosphate carboxylase oxygenase (Rubisco) and the regenerative capacity of ribulose diphosphate (RuBP) are uncovered to be essential for maintaining high photosynthetic CO_2_ fixation capacity. Previous studies revealed that three non-regulated enzymes, including fructose−1,6-bisphosphate aldolase (FBA or aldolase), sedoheptulose 1,7-bisphosphatase (SBPase), and transketolase (TK), had significantly higher control coefficient on photosynthesis than the other Calvin cycle enzymes, which indicated that they could limit photosynthetic rate and exert significant control over photosynthetic carbon flux other than Rubisco [41]. Particularly, FBA could catalyze the reversible conversion of DHAP and FBP and also catalyze DHAP and erythrose 4-phosphate (E4P) to sedoheptulose 1,7-bisphosphate (SBP). Thus, FBA is not only one of the key regulatory enzymes in the glycolysis pathway but also may lie in a vital strategic position to determine the carbon partitioning in the Calvin cycle, which made FBA probably an important candidate target of engineering to boost photosynthetic carbon CO_2_ fixation capacity [42]. 

*B. napus* is the second-largest source of vegetable oil crops and is cultivated around the world [33,34]. *B. napus* seeds contain oil, carbohydrates, and proteins as major storage reserves. In most seeds, glycolysis in plastids supplies carbon for fatty acid (FA) synthesis [43]. FBA, as a key enzyme in the glycolytic metabolism, provides precursors for amino acid and fatty acid synthesis [26]. To our best knowledge, there is not any report in the literature describing *FBA* family genes and their function in *B. napus* so far. In this study, we performed a comprehensive identification and characterization of the *FBA* gene family in *B. napus*. Compared to the number of *FBA* genes identified in other plant species, such as eight in *A. thaliana* [17], seven in rice [22], eight in tomato [19], and 21 in wheat [11], *B. napus* had more *FBA* genes, including 22 *BnaFBA* genes distributed in fifteen *B. napus* chromosomes and two random chromosomes. The *BnaFBAs* can be classified into two classes based on the functional domains contained, class-I and class-II. Enzyme kinetics analysis of aldolase 1 (class-I FBAs) and aldolase 2 (class-II FBAs) in *Escherichia coli* revealed that aldolase 1 and 2 hydrolyze fructose 1,6-bisphosphate by the aldol cleavage reaction [12]. Class-I BnaFBA proteins could form tetramer structures with high conserved catalytic residues of D-K-K-R-E-E-K-S-R that were homologous to those of rabbit FBA isozymes [44]. Class-II BnaFBA could form dimers with the active sites of H-E-H-H that corresponded to those of FBA isozymes in the *Thermus aquaticus* [45,46]. Eleven *BnaFBAs* of class-I and *BnaFBA9a* of class-II were predicted to be localized in chloroplast, while nine class-I *BnaFBAs* and *BnaFBA9b* of class-II were in the cytoplasm. This is consistent with the subcellular localization of their homologs in *A. thaliana* [17] and wheat [11]. Although AT1G18270 in *Arabidopsis* is the homolog of BnaFBA9a and BnaFBA9b, and it is annotated as ketose-bisphosphate aldolase class-II family protein; however, it contains fructose-bisphosphate aldolase class-II (pfam01116) domain and zinc-binding site and is further assigned as fructose-bisphosphate aldolase (NCBI Reference Sequence: NP_001117303.1). More accurately, AT1G18270 is fructose-bisphosphate aldolase class-II family protein. This inaccuracy might result from the homologous protein annotation in bacteria, such as *Variovorax paradoxus B4*. Now, the ketose-bisphosphate aldolase, class-II protein, was further assigned as fructose-1,6-bisphosphate aldolase, class-II in *Variovorax paradoxus B4* (GenBank: AGU50177.1). Therefore, *BnaFBA9a* and *BnaFBA9b* were classified as class-II *FBA* genes in *Brassica napus*. 

In contrast to the model plant *A. thaliana*, except for the paleo-polyploidization of alpha (α)-beta (β)-gamma (γ) WGD events, the *Brassica* species, such as *B. rapa* and *B. oleracea*, experienced an extra whole-genome triplication (WGT) event at approximately 15.9 Mya [47,48,49]. *B. napus* is a relatively new species of *Brassica* genus with a short history of post-Neolithic speciation (~7500 years) and domestication (~700 years) and a recent allotetraploid formed from hybridization between *B. rapa* and *B. oleracea* [35]. Therefore, *B. napus* has experienced five genome duplication events (3  ×  2  ×  2  ×  3  ×  2) at times during the evolution process. Gene duplication that resulted from whole-genome duplication (WGD), tandem duplication (TD), proximal duplication, transposed duplication, or dispersed duplication is one of the primary driving forces to the evolution of morphological and physiological diversity in plants [50]. Our results showed that *Brassica* species had an extended *FBA* gene family, containing extra copies of *FBA1*, *FBA2*, *FBA6*, and *FBA8*. Most gene duplications of the *FBA* gene family resulted from WGD (Table S2). Considering the collinear correlations and subgenome, transposed duplication and triplication events in the *FBA* family had contributed to this expansion prior to the speciation of *Brassica* species. WGD is often followed by intensive gene loss to adapt to continuously changing environments. Compared to *A. thaliana* and its two ancestors, *B. napus* species lost some copies of *FBA* genes (i.e., *FBA1*, *3*, 4, *5,* and *6*). The remaining duplicated or triplicated FBA genes identified might be conducive to the adaptation of *B. napus* to various adverse environments during speciation and domestication.

Genome resequencing provides an effective way to identify a large number of variations, which lay a foundation for further identification and functional validation of candidate genes contributed to important traits in crop plants, such as rice, tomato, and soybean [51,52,53]. Critical sequence polymorphisms across the gene and its flanking regions may reflect the evolutionary trends and breeding selection effects on the genes. Based on the resequencing of a worldwide collection of 991 *B. napus* germplasm accessions that released recently, we explored the pattern of genetic polymorphisms of the *BnaFBA* family genes. The results showed that the 22 *BnaFBAs* diversified in sequence polymorphism, with the polymorphism sites ranging from two to 130 (Table 3). The *BnaFBA* genes showed different levels of polymorphism. For example, *BnaFBA6*, *BnaFBA1c,* and *BnaFBA8d* with the highest levels of genetic variation polymorphism might have experienced weak selection pressure during the evolution process, whereas *BnaFBA2c*, *BnaFBA2d,* and *BnaFBA3b* were highly conserved after strong selection. Particularly, *BnaFBA6* harbored the highest levels of genetic variation, including two missense polymorphism sites with a frequency of 12.12% and 5.72%, respectively (Table S5). Moreover, a one-stop gained site with a frequency of 1.02% was also found in the CDS regions of the *BnaFBA6* gene. These results suggested that the *BnaFBA6* gene might be under the pseudogenization process. Furthermore, *BnaFBA* genes might have experienced balancing selection or population shrinkage. For example, six *FBA* family genes, including *BnaFBA8b*, *BnaFBA1b*, *BnaFBA1c*, *BnaFBA2e*, *BnaFBA1a,* and *BnaFBA2d*, harbored some selected signals and probably underwent breeding selection in the process of natural selection and domestication in *B. napus*. 

Fructose-1,6-bisphosphate aldolase (FBA) is a non-regulated enzyme in the Calvin cycle, whose activity is not regulated by effectors or posttranslational modification but by expressional regulation or protein degradation [42]. Recent studies using transgenic plants with reduced enzyme levels have revealed that FBAs play important roles in regulating carbon flux through the Calvin cycle. Elevated plastid aldolase activity accelerated RuBP regeneration and resulted in increased photosynthetic capacity, growth rate, and biomass yield in tobacco and cyanobacterium [27,54]. Increased activity of FBA in *Anabaena sp*. strain PCC 7120 stimulated photosynthetic yield [55]. Generally, the expression levels of *BnaFBA* family genes were higher in the overground tissues than that in the underground. In *B. napus*, some members of the FBA family, such as *BnaFBA5a*, *BnaFBA5b*, *BnaFBA2b*, *BnaFBA2c,* and *BnaFBA2d*, showed higher expression levels in mature leaves and the top of stems than that in young leaves and the base of stems. Notably, *BnaFBA* genes were preferentially highly expressed in the photosynthetic tissues and stages, particularly leaves and silique wall in *B. napus*. For example, during the development of silique, *BnaFBA5a* and *BnaFBA5b* exhibited the highest expression levels at 10 days after DAP, while *BnaFBA1a*, *BnaFBA2a,* and *BnaFBA2b* had highest expression levels at 15 days after DAP in the silique wall. Besides, *BnaFBA1a*, *BnaFBA2a*, *BnaFBA2b*, *BnaFBA2c*, *BnaFBA2d*, *BnaFBA2e,* and *BnaFBA2f* showed the highest expression levels at four weeks after DAP in the seed tissues when seed fill begins with rapid embryo growth, oil biosynthesis, and protein accumulation [56]. In addition, *BnaFBA3a*, *BnaFBA3b*, *BnaFBA8c,* and *BnaFBA8d* also showed the highest expression levels at 25 days in the embryo tissues. Thus, the members of *BnaFBAs* that have a strong correlation with photosynthetic events are potential targets for engineering to improve photosynthetic capacity in the future, which would be further investigated in our next study.

Previous studies revealed *FBA* family genes also involved in plant defense and response to various biotic and abiotic stresses, such as cold and heat stress [17], salt stress [21], drought stress [21], water-deficit stress [30], stress with *Rhizoctonia solani* Kuhn [31], and high light acclimation stress [32]. Here, we found that the members of the *BnaFBA* family showed diverse expression patterns in response to *Sclerotinia sclerotiorum* infection and drought stress in *B. napus*. For example, *BnaFBA1a/b/c*, *BnaFBA2a/b/c/d/e/f*, *BnaFBA5a/b,* and *BnaFBA9b* were down-regulated, while *BnaFBA3a/b* and *BnaFBA8a/b/c/d/e/f* were up-regulated compared to the control in the leaves at 24 h after *Sclerotinia sclerotiorum* infection. Under drought stress, the expression levels of 10 *BnaFBA* genes (*BnaFBA1a/c*, *BnaFBA2a/b/c/d/e/f,* and *BnaFBA5a/b*) decreased in the leaves and five *BnaFBA* genes (*BnaFBA1b*, *BnaFBA2b/d/e/f*) lowered in roots. Besides, we applied salt, heat, and drought stresses on *B. napus* seedlings to explore the expression changes of *BnaFBAs*. The expression of the *BnaFBAs* selected was induced by the three stresses. The expression levels of *BnaFBA2a*, *BnaFBA5b,* and *BnaFBA9a* were down-regulated, while *BnaFBA8a* was up-regulated compared to the control after treatments. In addition, *FBA* family genes were reported to play roles in response to phytohormones, such as ABA [17] and GA [29]. Various *cis*-acting regulatory elements related to phytohormone responsive elements were observed in the promoter regions of *BnaFBA* genes in *B. napus*, including ABRE, AuxRE, ERE, GARE, MeJARE, and SARE, indicating that the expression levels of *BnaFBA* family genes might be affected by ABA, IAA (indolylacetic acid), ethylene, GA, MeJA, and SA. Furthermore, our results showed that *BnaFBAs,* such as *BnaFBA2a/b/c/d/e/f*, could be also induced by strigolactones (SLs), a new class of plant hormones playing functional roles in the development of root.

In wheat, some FBA genes showed close correlations in expression patterns and could be classified into different clusters, such as *TaFBA1/2/3*, *TaFBA14/15/17*, and *TaFBA19/20/21* [11]. Similarly, based on the co-regulatory networks of *BnaFBAs*, the 22 *BnaFBA* family genes could be divided into three gene clusters. Strong positive correlations between the expression levels were observed among the members within each gene cluster of *BnaFBAs*. Meanwhile, only a few significant negative correlations appeared between minority members of cluster 1 and cluster 2. Cluster 3, consisting of *BnaFBA9a/b* of class-II, was independent among the three clusters. The results suggested that the *BnaFBA* genes might have functional redundancy within gene clusters and functional diversification among gene clusters in *B. napus*.

In summary, we performed a genome-wide identification of *FBA* family genes in *B. napus*, as well as *B. rapa* and *B. oleracea*, and further investigated their gene structures and phylogenetic relationships. The *cis*-acting regulatory elements in the promoter regions, natural variations, and expression patterns of *BnaFBAs* in different tissues or under treatments were analyzed. Our findings provided useful information regarding *FBA* family genes in *B. napus*. Remarkably, we found that some members of the *FBA* family that underwent breeding selection and were highly expressed in leaves and silique wall probably had positive roles in the promotion of photosynthetic capacity in *B. napus*. These *BnaFBA* genes might be utilized in the development and selection of high-yield *B. napus* cultivars.

## 4. Materials and Methods

### 4.1. Identification and Property Analysis of BnaFBA Genes

The genome sequence, annotation, and protein datasets of *B. napus* var. Darmor-*bzh*, *B. rapa* var. Z1, and *B. oleracea* var. HDEM were obtained from the GENOSCOPE database (http://www.genoscope.cns.fr/brassicanapus/ and http://www.genoscope.cns.fr/externe/plants/). The glycolytic domain (PF00274) and fructose-bisphosphate aldolase class-II domain (PF01116) from the Pfam database (http://pfam.xfam.org/) were applied as queries to search against local *B. napus* protein sequence dataset using HMMER (version 3.2.1, HHMI, Chevy Chase MD, USA) with *E*-value setting at 1e-5 [57]. Then, for further confirmation of BnaFBA proteins, the sequences of predicted BnaFBA proteins were searched against all the annotated proteins of *A. thaliana* and wheat (*Triticum aestivum* L.) using BLASTP (version 2.2.26, Bethesda, MD, USA) with *E*-value < 1 × 10^-5^. The putative BnaFBAs with best hits of the *A. thaliana* and wheat FBA proteins remained and were further deployed to determine the fructose-bisphosphate aldolase domains by using the Pfam and SMART databases (http://smart.embl-heidelberg.de/). The molecular weight (MW), isoelectric point (pI), and grand average of hydropathy (GRAVY) of each BnaFBA were calculated using the ProtParam tool (http://web.expasy.org/protparam/).

### 4.2. Cis-Acting Regulatory Elements and Subcellular Localization Analysis

The 1.5 kb promoter sequence upstream from the transcription start site of each *BnaFBA* gene was extracted from the *B. napus* genome sequence and used to predict *cis*-acting regulatory elements by PlantCARE [58]. The subcellular localization of each BnaFBA was predicted by Plant-mPLoc (http://www.csbio.sjtu.edu.cn/bioinf/plant/) [59].

### 4.3. Structure and Chromosomal Localization Analysis

The gene structures of the *BnaFBA* genes were inferred by aligning their coding sequences to the *B. napus* genomic sequence. Then, a schematic map of the exon-intron structure of each *BnaFBA* gene was drawn by Gene Structure Display Server 2.0 (http://gsds.cbi.pku.edu.cn/) [60]. Three-dimensional (3D) protein models of the BnaFBAs were generated using SWISS-MODEL [61,62]. The physical chromosomal locations of *BnaFBAs* were obtained from the *B. napus* genome annotation information. The graphical representation of the *BnaFBA* genes on chromosomes was plotted using R software with RIdeogram package (https://github.com/TickingClock1992/RIdeogram) [63].

### 4.4. Multiple Alignments and Phylogenetic Analysis

Multiple alignments of FBA protein sequences from *B. napus*, *B. rapa*, *B. oleracea*, *A. thaliana,* and wheat were performed using MUSCLE (version 3.8, Hinxton, Cambridge, UK) with default parameters [64]. The phylogenetic tree was generated using MEGA7.1 with the neighbor-joining method, and the robustness of each node in the tree was determined using 1000 bootstrap replicates [65].

### 4.5. Synteny and Duplicate Gene Analysis

The genomic collinearity between all pairwise combinations of *B. napus*, *B. rapa*, *B. oleracea*, and *A. thaliana* genomes was analyzed using MCScanX (version Nov. 11, 2013, Athens, GA, USA) with the default parameters [66]. Then, the syntenic relationships of *BnaFBAs*, *BraFBAs*, *BolFBAs,* and *AtFBAs* were determined according to the genomic collinearity between pairwise genomes. The syntenic map was illustrated by CIRCOS (version 0.69-9, BCGSC, Vancouver, BC, Canada) software [67,68]. The duplicated gene pairs and the modes of gene duplication were identified among *FBA* genes using DupGen_finder (https://github.com/qiao-xin/DupGen_finder) in *B. napus*, *B. rapa*, *B. oleracea*, and *A. thaliana* [69].

### 4.6. Variations Analysis and Principal Component Analysis

The publically available genome resequencing datasets of 991 *B. napus* germplasm accessions were downloaded from the National Center of Biotechnology Information (NCBI) under SRP155312 [39]. The sequencing reads for each accession were mapped to *B. napus* var. Darmor-*bzh* reference genome using the MEM algorithm of Burrows–Wheeler Aligner (version 0.7.17, Hinxton, Cambridge, UK) [70]. The mapping results were sorted using SAMTOOLS (version 1.1, Hinxton, Cambridge, UK) [71]. The duplicated reads were marked with PICARD (version 2.0.1, Broad Institute, Cambridge, MA, USA) [72]. Variations, including SNPs and InDels, were called using the HaplotypeCaller module in GATK (version 4.1.3.0, https://github.com/broadinstitute/gatk/) for each accession. SNP and InDels annotation was performed using ANNOVAR (version 2018Apr16, Philadelphia, PA, USA) software based on the annotation of *B. napus* var. Darmor-*bzh* genome [73]. Principal component analysis (PCA) was performed using the R software with the ggbiplot package (https://github.com/vqv/ggbiplot).

### 4.7. Expression Analysis of BnaFBA Genes

The publically available RNA-seq dataset of 12 different tissues (sepal, pistil, stamen, ovule, pericarp, blossomy pistil, wilting pistil, root, flower, leaf, silique wall, and stem) collected from different stages of the growth (BioProject ID: PRJNA394926) [36], RNA-seq dataset of leaf and root tissues under drought stress (BioProject ID: PRJNA256233) [74], RNA-seq dataset of seeds across four phases of the development (BioProject ID: PRJNA311067) [56], RNA-seq dataset of stems and leaves after *Sclerotinia sclerotiorum* infection (BioProject ID: PRJNA321917) [75], and time-series RNA-seq dataset of roots under a synthetic analog of strigolactones (rac-GR24) treatments (BioProject ID: PRJNA484313) [76] were downloaded from the NCBI SRA database, and further used as main sources to perform gene expression profiling of *BnaFBA* genes in *B. napus*. The transcriptome reads were mapped to *B. napus* var. Darmor-*bzh* reference genome using HISAT2 (version 2.1.0, Baltimore, MD, USA) with the default settings [77]. The read counts per gene were generated by featureCounts [78]. Fragments per kilobase of exon per million fragments mapped (FPKM) was used for the quantification of gene expression. The clustered heatmaps were visualized with expression levels (log2) of *BnaFBA* genes by R software using the pheatmap function package (https://cran.r-project.org/web/packages/pheatmap/).

### 4.8. Plant Materials and Treatments

Rapeseed seeds were germinated on a filter paper saturated with distilled water in darkness at 22 °C for two days. Then, the seedling plants were transferred to a 4 L hydroponic system containing continuously aerated 1/2 Murashige and Skoog (MS) liquid solution (pH 5.8, without agar and sugar) and grown in an incubator under a photosynthetic flux of 160 μmol photons m^−2^ s^−1^ and a humidity of about 50% (16 h light at 25 °C/8 h darkness at 22 °C). The 1/2 MS liquid solution was changed once every two days. After three weeks, the seedlings were transferred to a new 1/2 MS liquid solution (pH 5.8, without agar and sugar) for different stress treatments. For salt, heat, and drought stress treatments, seedlings were exposed to 1/2 MS solution (pH 5.8, without agar and sugar) containing 250-mM NaCl, 40 °C conditions, and 20% (*w/v*) polyethylene glycol (PEG), respectively. Seedlings exposed to 1/2 MS solution at 22 °C were used as controls. Leaf samples were collected 3 days after each treatment. All collected samples were immediately frozen in liquid nitrogen and stored at −70 °C for further analysis.

### 4.9. RNA Isolation and Quantitative Real-Time Polymerase Chain Reaction (qRT-PCR) Analysis

Total RNAs were extracted from each sample using an RNA extraction kit (Takara, Dalian, China) following the manufacturer’s procedure. Two micrograms of total RNA was used to synthesize the first-strand cDNA using the Prime Script RT reagent Kit (Takara, Dalian, China) according to the manufacturer’s protocol. Real-time quantitative PCR was performed using 2 mL of cDNA in a 20 mL reaction volume using an SYBR Green PCR kit (GeneCopoeia Inc., Rockville, MD, USA) with ViiATM 7 Dx platform (ABI, Los Angeles, CA, USA). The qRT-PCR reaction condition was as follows: 95 °C for 5 min, 40 cycles at 95 °C for 30 s, 55 °C for 30 s, and 72 °C for 30 s. Gene-specific primers were designed and listed in Supplementary Materials (Table S6). The relative expression levels of these genes were analyzed by the 2^−^^△△*C*t^ method. The *BnaTMA7* gene (*BnaC05g11560D*), which exhibited stable expression in different/same tissues under various experimental conditions, was used as an internal control [79]. All qRT-PCR reactions were assayed in triplicates.

### 4.10. Pearson Correlation Analysis

On the basis of the RNA-seq results, the Pearson correlation coefficients (PCCs) and *p*-value of the expression levels of *BnaFBA* gene pairs were calculated using R software with cor and cor.test function packages, respectively. The correlation heatmap was generated by R software with the corrplot function package. The gene co-regulatory networks were constructed by Cytoscape (version 3.1, Seattle, WA, USA) based on the PCCs of *BnaFBA* gene pairs with a *p*-value ≤ 0.05 [80].

## Figures and Tables

**Table 1:** Summary Information on *FBA* Family Genes in *B. Napus*.

Gene Name	Locus Name	Gene Location ^1^	Transcript Length (bp)	Protein Length (aa)	MW (kDa)	pI	GRAVY	Domain ^2^ (Start-End aa)	Homolog of *Arabidopsis*	Classifi-cation	Subcellular Localization
*BnaFBA1a*	BnaA04g12130D	chrA04:10353159–10354905:−	1173	390	42.2	6.92	−0.194	PF00274 (55–390)	*AT2G21330* (*AtFBA1*)	I	Chloroplast
*BnaFBA1b*	BnaC04g33570D	chrC04:35146154–35147860:+	1173	390	42.16	6.92	−0.19	PF00274 (55–390)	*AT2G21330* (*AtFBA1*)	I	Chloroplast
*BnaFBA1c*	BnaC08g35820D	chrC08:33457869–33459923:+	1197	398	42.81	6.78	−0.155	PF00274 (54–398)	*AT2G21330* (*AtFBA1*)	I	Chloroplast
*BnaFBA2a*	BnaA08g16820D	chrA08:13562506–13564430:+	1152	383	41.3	5.7	−0.179	PF00274 (44–383)	*AT4G38970* (*AtFBA2*)	I	Chloroplast
*BnaFBA2b*	BnaC03g60280D	chrC03:49437707–49439536:−	1152	383	41.33	5.7	−0.185	PF00274 (44–383)	*AT4G38970* (*AtFBA2*)	I	Chloroplast
*BnaFBA2c*	BnaC07g47470D	chrC07:44670819–44672806:+	1182	393	42.58	6.87	−0.196	PF00274 (54–393)	*AT4G38970* (*AtFBA2*)	I	Chloroplast
*BnaFBA2d*	BnaA06g37230D	chrA06:24314148–24316076:+	1182	393	42.64	7.61	−0.208	PF00274 (54–393)	*AT4G38970* (*AtFBA2*)	I	Chloroplast
*BnaFBA2e*	BnaAnng40850D	chrAnn_random:46872917–46874555:+	951	317	34.18	6.77	−0.036	PF00274 (54–306)	*AT4G38970* (*AtFBA2*)	I	Chloroplast
*BnaFBA2f*	BnaC01g00070D	chrC01:20706–22753:−	1200	399	42.95	6.07	−0.137	PF00274 (44–399)	*AT4G38970* (*AtFBA2*)	I	Chloroplast
*BnaFBA3a*	BnaA02g27140D	chrA02:20044496–20046087:+	1170	389	42.3	8.47	−0.263	PF00274 (45–389)	*AT2G01140* (*AtFBA3*)	I	Chloroplast
*BnaFBA3b*	BnaC02g33660D	chrC02:36012928–36014598:−	1170	389	42.28	8.47	−0.257	PF00274 (45–389)	*AT2G01140* (*AtFBA3*)	I	Chloroplast
*BnaFBA5a*	BnaA01g15640D	chrA01:8017642–8018970:+	1077	358	38.23	6.38	−0.072	PF00274 (11–358)	*AT4G26530* (*AtFBA5*)	I	Cytoplasm
*BnaFBA5b*	BnaC01g18640D	chrC01:12962252–12967551:+	2613	870	95.97	6.82	−0.107	PF00485 (52–237); PF14681 (266–458); PF00274 (523–870)	*AT4G26530* (*AtFBA5*)	I	Cytoplasm
*BnaFBA6*	BnaAnng07310D	chrAnn_random:7183202–7186036:+	1032	343	36.91	5.8	−0.189	PF00274 (11–338)	*AT2G36460* (*AtFBA6*)	I	Cytoplasm
*BnaFBA8a*	BnaA09g33290D	chrA09:24581809–24583398:−	1077	358	38.45	6.28	−0.197	PF00274 (11–358)	*AT3G52930* (*AtFBA8*)	I	Cytoplasm
*BnaFBA8b*	BnaC08g24100D	chrC08:26197819–26199427:-	1077	358	38.45	6.28	−0.197	PF00274 (11–358)	*AT3G52930* (*AtFBA8*)	I	Cytoplasm
*BnaFBA8c*	BnaC06g14270D	chrC06:17065486–17066935:−	1077	358	38.42	6.22	−0.177	PF00274 (11–358)	*AT3G52930* (*AtFBA8*)	I	Cytoplasm
*BnaFBA8d*	BnaA07g15900D	chrA07:13618484–13619930:−	1077	358	38.42	6.22	−0.177	PF00274 (11–358)	*AT3G52930* (*AtFBA8*)	I	Cytoplasm
*BnaFBA8e*	BnaA04g05120D	chrA04:3768229–3769737:+	1077	358	38.49	6.28	−0.218	PF00274 (11–358)	*AT3G52930* (*AtFBA8*)	I	Cytoplasm
*BnaFBA8f*	BnaCnng50220D	chrCnn_random:49773855–49775405:+	1077	358	38.49	6.28	−0.218	PF00274 (11–358)	*AT3G52930* (*AtFBA8*)	I	Cytoplasm
*BnaFBA9a*	BnaA06g12420D	chrA06:6440041–6449829:−	4143	1380	147.66	5.82	0.067	PF01116 (1104–1379)	*AT1G18270* (ketose-bisphosphate aldolase class-II family protein)	II	Chloroplast
*BnaFBA9b*	BnaC05g14020D	chrC05:8111222–8120981:−	4149	1382	148	5.75	0.076	PF01116 (1106–1381)	*AT1G18270* (ketose-bisphosphate aldolase class-II family protein)	II	Cytoplasm

^1^ Chromosome: start position-end position: strand, (–) means antisense strand of chromosome, (+) means positive-sense strand of chromosome. ^2^ Glycolytic, fructose-bisphosphate aldolase class-I (PF00274); F_bP_aldolase, fructose-bisphosphate aldolase class-II (PF01116); Uracil phosphoribosyltransferase, UPRTase (PF14681); PRK, phosphoribulokinase / uridine kinase family (PF00485); AP2, apetala 2 (PF00847).

**Table 2:** *Cis*-Acting Elements in the Promoter Region of 22 *BnaFBA* Genes.

Gene	*Cis*-Acting Elements ^1^														
	ABRE	ARE	AuxRE	Circadian	DIRE	DRE	DSRE	ERE	GARE	HSRE	LRE	LTRE	MeJARE	SARE	WRE
*BnaFBA1a*	13	3	1					1			26	1	2		
*BnaFBA1b*	11	7			2		1			2	16	2	8	2	
*BnaFBA1c*	5	3	1				1	1			21		4	1	
*BnaFBA2a*	4	1	2				1	3	1		16	1	10		
*BnaFBA2b*	4		2				1	3	2		18		12		
*BnaFBA2c*	4	1	2			1		1	2		14		4		
*BnaFBA2d*	4	3	3					1	2		14		4		
*BnaFBA2e*	4	6						1	1	2	13	1		1	
*BnaFBA2f*	12	4						1		1	19	2	4		
*BnaFBA3a*	2			2			1	1	1	1	10	1		2	
*BnaFBA3b*		3	1		2			1	2	1	9		2		
*BnaFBA5a*	5	1		1	2		2	1	1	1	13		2		
*BnaFBA5b*	1	3				1			2	1	10		10		1
*BnaFBA6*	1				1	3		1	2	1	13		4	1	
*BnaFBA8a*	7	5				2	1	2		1	16	1	4	1	
*BnaFBA8b*	1	2				1	1	2	1	1	10		2		
*BnaFBA8c*	2	9			1			4		2	8	1			1
*BnaFBA8d*	2	4		1	1		1			5	9	1		2	
*BnaFBA8e*	4	2	1	1			1	3		1	15		4	1	
*BnaFBA8f*	3	3	1	1						1	11		8		
*BnaFBA9a*	15	2	1		1		1	1		1	23		10	1	1
*BnaFBA9b*	3	3				1	1	1			13	1	2		

^1^ Abscisic acid-responsive element (ABRE), Anoxic-responsive element (ARE), Auxin-responsive element (AuxRE), Circadian-responsive element (Circadian), Damage-responsive element (DRE), Defense- and stress-responsive element (DSRE), Drought-responsive element (DIRE), Ethylene-responsive element (ERE), Gibberellin-responsive element (GARE), Heat stress-responsive element (HSRE), Light-responsive element (LRE), Low-temperature-responsive element (LTRE), MeJA-responsive element (MeJARE), Salicylic acid-responsive element (SARE), and Wound-responsive element (WRE).

**Table 3:** Summary of Polymorphic Sites of the 22 *BnaFBA* Genes.

Gene	CDS Length (bp)	Sample Size	No. of Polymorphic Sites ^1^	Sequence Variation Ratio (%)	Nucleotide Diversity		Tajima’s D ^4^
					π/bp ^2^	θ_w_/bp ^3^	
*BnaFBA1a*	1173	401	11	0.94	0.00376	0.00187	2.28214 *
*BnaFBA1b*	1173	469	7	0.60	0.00286	0.00155	2.56152 **
*BnaFBA1c*	1197	198	60	5.01	0.01299	0.00762	2.14201 *
*BnaFBA2a*	1152	163	23	2.00	0.00657	0.00394	1.45126
*BnaFBA2b*	1152	505	10	0.87	0.00279	0.00211	0.98657
*BnaFBA2c*	1182	780	2	0.17	0.00113	0.00092	1.89306
*BnaFBA2d*	1182	727	3	0.25	0.00145	0.00098	1.9818 *
*BnaFBA2e*	951	289	10	1.05	0.0044	0.00239	2.43882 *
*BnaFBA2f*	1200	552	21	1.75	0.00509	0.00298	1.90478
*BnaFBA3a*	1170	249	34	2.91	0.00894	0.00665	0.81257
*BnaFBA3b*	1170	210	5	0.43	0.00057	0.00054	0.17874
*BnaFBA5a*	1077	307	21	1.95	0.00783	0.00535	1.49221
*BnaFBA5b*	2613	228	42	1.61	0.0053	0.00349	1.63186
*BnaFBA6*	1032	132	130	12.60	0.04158	0.02255	1.99668
*BnaFBA8a*	1077	379	29	2.69	0.00959	0.00706	1.32819
*BnaFBA8b*	1077	529	29	2.69	0.01251	0.0056	3.68226 ***
*BnaFBA8c*	1077	726	5	0.46	0.00241	0.00143	1.43186
*BnaFBA8d*	1077	251	38	3.53	0.01206	0.00807	1.33435
*BnaFBA8e*	1077	234	31	2.88	0.0108	0.00598	1.93228
*BnaFBA8f*	1077	429	12	1.11	0.0033	0.00208	1.81155
*BnaFBA9a*	4143	100	79	1.91	0.00467	0.00355	0.97966
*BnaFBA9b*	4149	43	86	2.07	0.00673	0.00511	1.19193

^1^ polymorphic includes the SNPs (single nucleotide polymorphisms) and Indels (insertions and deletions). ^2^ π, average nucleotide differences per site between the two sequences. ^3^ θ_w_, Watterson estimator. ^4^ Tajima’s D, test for neutral selection (*: significant at *p* < 0.01; **: significant at *p* < 0.001; ***: significant at *p* < 0.0001). CDS, coding sequence.

## Supplementary Materials

Supplementary materials can be found at https://www.mdpi.com/1422-0067/20/22/5749/s1.

## Abbreviations

ABA	Abscisic acid
IAA	Indolylacetic acid
MeJA	Methyl Jasmonic acid
GA	Gibberellin
SA	Salicylic acid
SLs	Strigolactones
EDTA	Ethylene diamine tetraacetic acid
CDS	Coding sequence
GRAVY	Grand average of hydropathy
FPKM	Fragments per kilobase of exon per million fragments mapped
PCCs	Pearson correlation coefficients
SNP	Single nucleotide polymorphism
Indels	Insertions and deletions
MW	Molecular weight
pI	Isoelectric point
aa	Amino acid
bp	Base pair
kb	Kilobase
Mb	Megabase
kDa	Kilodalton
