# Supplementary material for: Genome-Wide Identification and Characterization of FBA Gene Family in Polyploid Crop Brassica napus
Source: Int J Mol Sci. 2019 Nov 15;20(22):5749. doi: 10.3390/ijms20225749 (PMC6888112; doi:10.3390/ijms20225749)
Supplement: Supplementary file 1 [file ijms-20-05749-s001.zip › ijms-644922-supplementary-20191114/ijms-644922-supplementary.pdf]

Table S1. Summary information of *FBA* gene family in *B. rapa* and *B. oleracea*

Table S2. Gene duplication of *FBA* gene family in *B. napus*, *B. rapa*, *B. oleracea* and *A. thaliana*

Table S3. Annotation of *cis*-acting regulatory elements in the promoters of *BnaFBA* genes.

Table S4. Variations statistic of *BnaFBA* genes in *B.napus*.

Table S5. Variations in the coding regions of the 22 *BnaFBA* genes.

Table S6. List of primers for qRT-PCR.

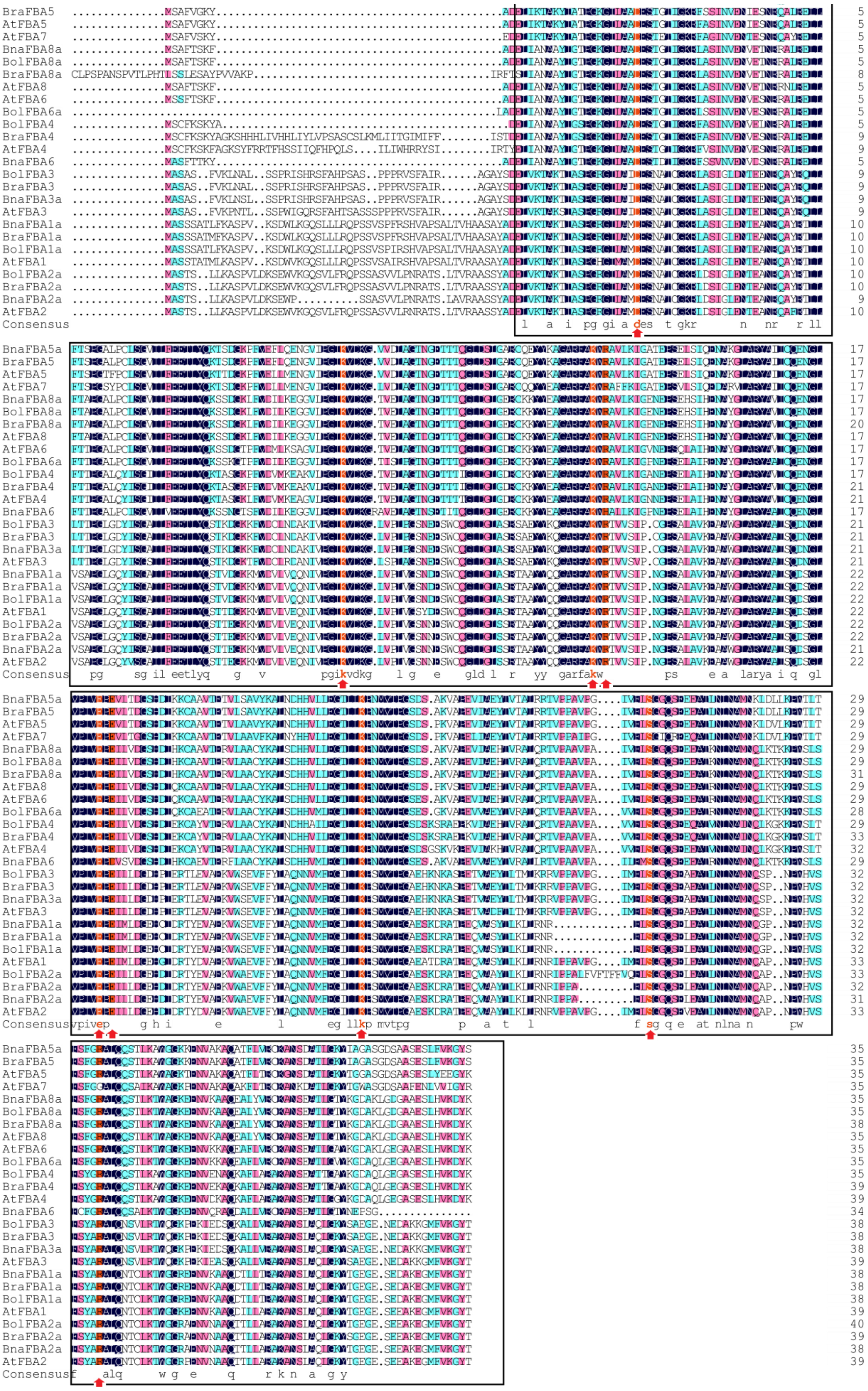

Figure S1. The alignment of class-I FBA protein sequences among *B. napus* and other species. The catalytic residues are indicated in red arrows. The black frames represent the fructose-bisphosphate aldolase class-I (PF00274) domain. *AtFBA*, *BnaFBA*, *BolFBA*, *BraFBA* indicated the *FBA* genes from *A. thaliana*, *B. napus*, *B. oleracea* and *B. rapa*, respectively.

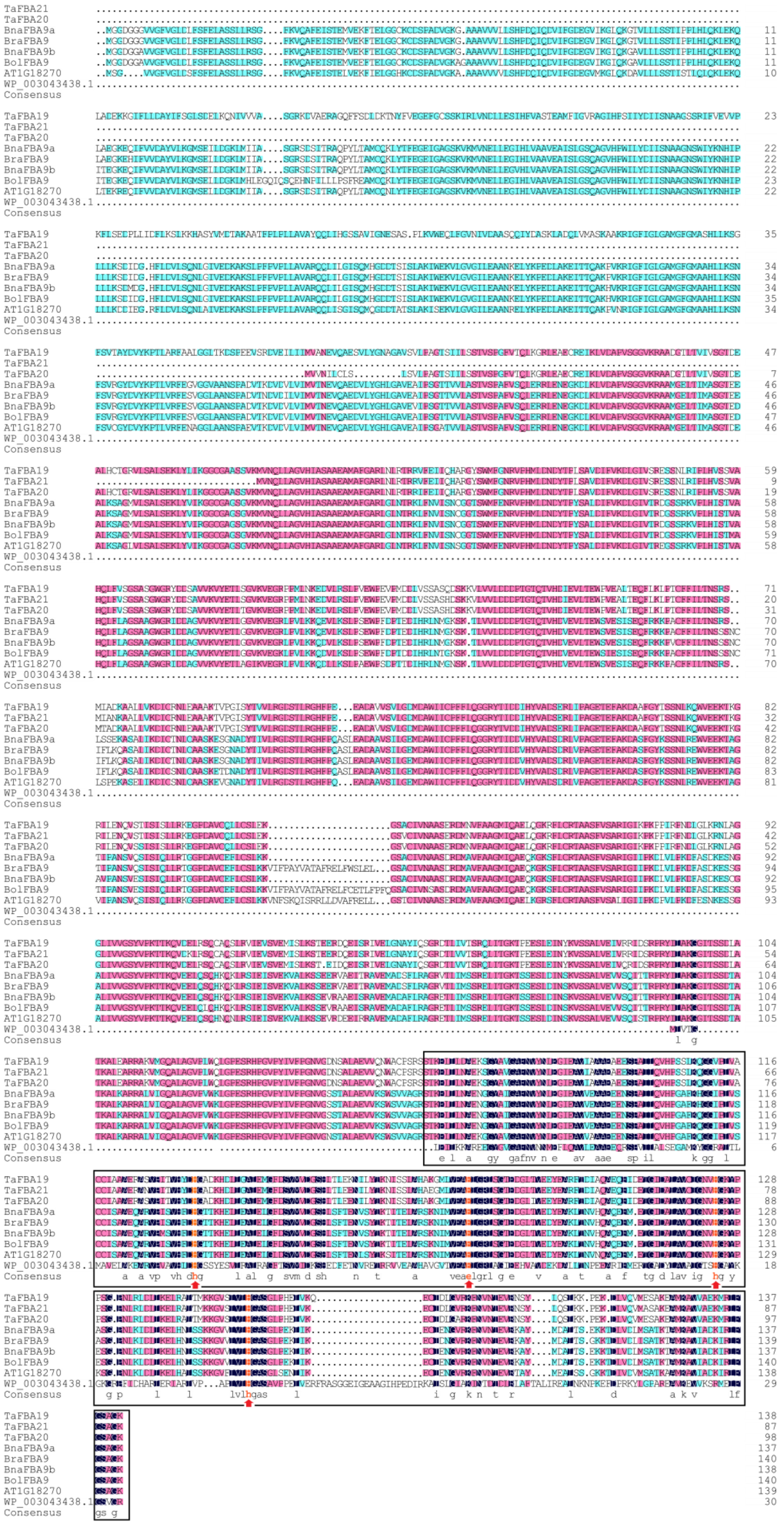

Figure S2. The alignment of class-II FBA protein sequences among *B. napus* and other species. The activity sites are indicated in red arrows. The black frames represent the fructose-bisphosphate aldolase class-II (PF01116) domain. *TaFBA*, *BnaFBA*, *BolFBA*, *BraFBA* indicated the *FBA* genes from *T. Aestivum*, *B. napus*, *B. oleracea* and *B. rapa*, respectively. *WP_003043438.1* was class II fructose-1,6-bisphosphate aldolase from *Thermus aquaticus*.

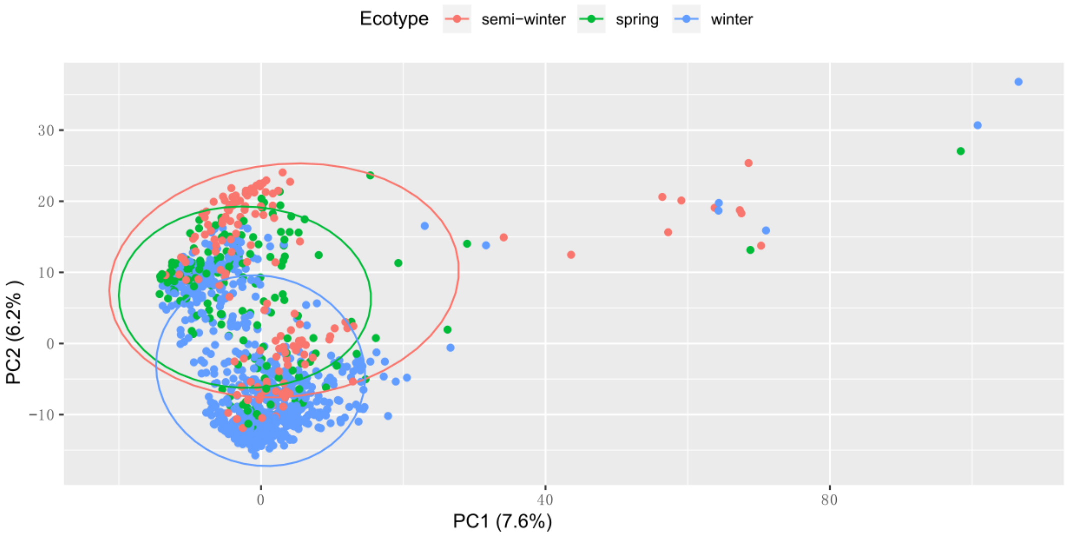

Figure S3. PCA plot of the first two components (PC1 and PC2) of the 991 accessions.
